# Wilhelm Keller MD (1818–1877) and the emergence of xenobiochemistry

**DOI:** 10.1177/09677720241273694

**Published:** 2024-08-16

**Authors:** Stephen Mitchell, Rosemary Waring

**Affiliations:** 1 Faculty of Medicine, 4615 Imperial College London, London, UK; 2 School of Biosciences, 1724 University of Birmingham, Birmingham, UK

**Keywords:** Wilhelm Keller, drug, metabolism, xenobiochemistry, hippuric acid

## Abstract

Although there had been many previous inklings, the field of xenobiotic metabolism (as we know it today) began with an experiment reported in the 1841 literature proclaiming that the ingestion of benzoic acid led to the subsequent excretion of hippuric acid in human urine. A metabolic transformation undertaken by a living organism. One worker involved in the early stages of this discovery was Wilhelm Keller, although very little information about him is readily available. Hopefully, this article will go some way to counter this dearth and also highlight Keller's pioneering contribution in the development of the fields of drug metabolism and xenobiochemistry.

## Introduction

That foods and their constituents were in some way altered as they travelled through the human body was self-evident from very early times. A familiar example is asparagus that has been known for centuries to impart a strong odour of rotten cabbage to the urine which bears no resemblance whatsoever to the aroma of the original vegetable.^1^ If nutrients were altered in some way, then why not other ingested components? William Cullen (1710–1790), Thomas Percival (1749–1804) and Antoine-François de Fourcroy (1755–1809), amongst others, speculated that medications, usually as mixtures, might also undergo such decomposition or alteration within the body. However, these early observations resulted from the ingestion of a variable and uncontrolled mixture of substances. The dosing of a measured amount of a pure single chemical and the identification and quantification of the excreted biotransformation products had to await the appropriate developments in medicine, physiology and chemistry.

Although there was a growing interest and wonderment in the materials excreted in the urine, organic compounds produced with the aid of the ‘vital force’ within living organism,^2^ the first deliberate experiment where a pure substance was administered to a human in order to identify and quantitate its subsequent excretory products was undertaken by Alexander Ure (1808–1866) in 1840 and published in the literature a year later.^3,4^ Ure was a Scottish physician and surgeon, educated at Glasgow and Edinburgh Universities, and worked in London both as a private practitioner and at several institutions including St Mary's Hospital, Paddington. He was examining the idea of introducing chemicals into the body to combine with endogenous materials in order to dissolve or prevent the insoluble deposition of bladder stones and gouty concretions. During his medical researches he administered benzoic acid and identified hippuric acid in the subsequently voided urine. He made several predictions concerning its formation, including the involvement of uric acid (lithic acid), even invoking the use of benzoic acid as a potential treatment, but unfortunately these forecasts were incorrect.^3,4^

Two other workers, Bouis and Garrod, subsequently undertook similar experiments. Dominique François Raymond Jules Bouis (1822–1886), was a French pharmacist from Perpignon who had attended the University of Montpellier and spent time working in the laboratory of Jean Baptiste André Dumas (1800–1884) in the Rue Cuvier before receiving a doctorate of science and becoming professor of chemistry/toxicology at the Paris School of Pharmacy.^5–7^ Alfred Baring Garrod (1819–1907) was an English physician who undertook training at the Ipswich Hospital before receiving a medical doctorate from the North London Hospital (now University College Hospital). Following work in the fields of rheumatoid arthritis and gout he became professor of materia medica and therapeutics at King's College Hospital, London and ‘physician extraordinary’ to Queen Victoria.^8,9^ Both of these individuals successfully repeated Ure's work but they did not offer any further insights except perhaps Garrod, who by finding minute crystals of uric acid contaminating his crude hippuric acid, offered an explanation for its lack of presence in the treated urine of Ure's experiment, which had led Ure to the erroneous assumption of the involvement of uric acid in the reaction.^10^ Around this seminal observation there developed a fervour of activity, and Keller was about to enter into its midst.

## Wilhelm Keller

Wilhelm Karl Christian Keller (1818–1877) was born on 2^nd^ March 1818 in Griesheim, within the Grand Duchy of Hesse, (Darmstadt-Dieburg) western Germany. His parents, August Heinrich Keller (1776–1825) from Griesheim and Helena Juliana Keller (née, Fürste/Föerster/Forsterine) (1781–1826) from Hessen, were married in Griesheim on 6^th^ May 1815. His forebears had resided in Germany from at least the end of the sixteenth century and had been proprietors of the ‘*Zum weißen Schwan*’ (the White Swan) in Griesheim since 1645.^11^ One of Wilhelm's siblings, an elder brother August Heinrich Keller (1816–1884), trained with Franz Heinrich Köhler (1806–1871) as a book merchant in Stuttgart before taking over the renowned Frankfurt bookstore of Siegmund Schmerber (1801–1840) during July 1841. He married Maria Friederika Eyssen (1823–1876) in 1852 and in the mid-1850s acquired the associated printing house with himself becoming a successful publishing bookseller.^12^

**Figure 1.**
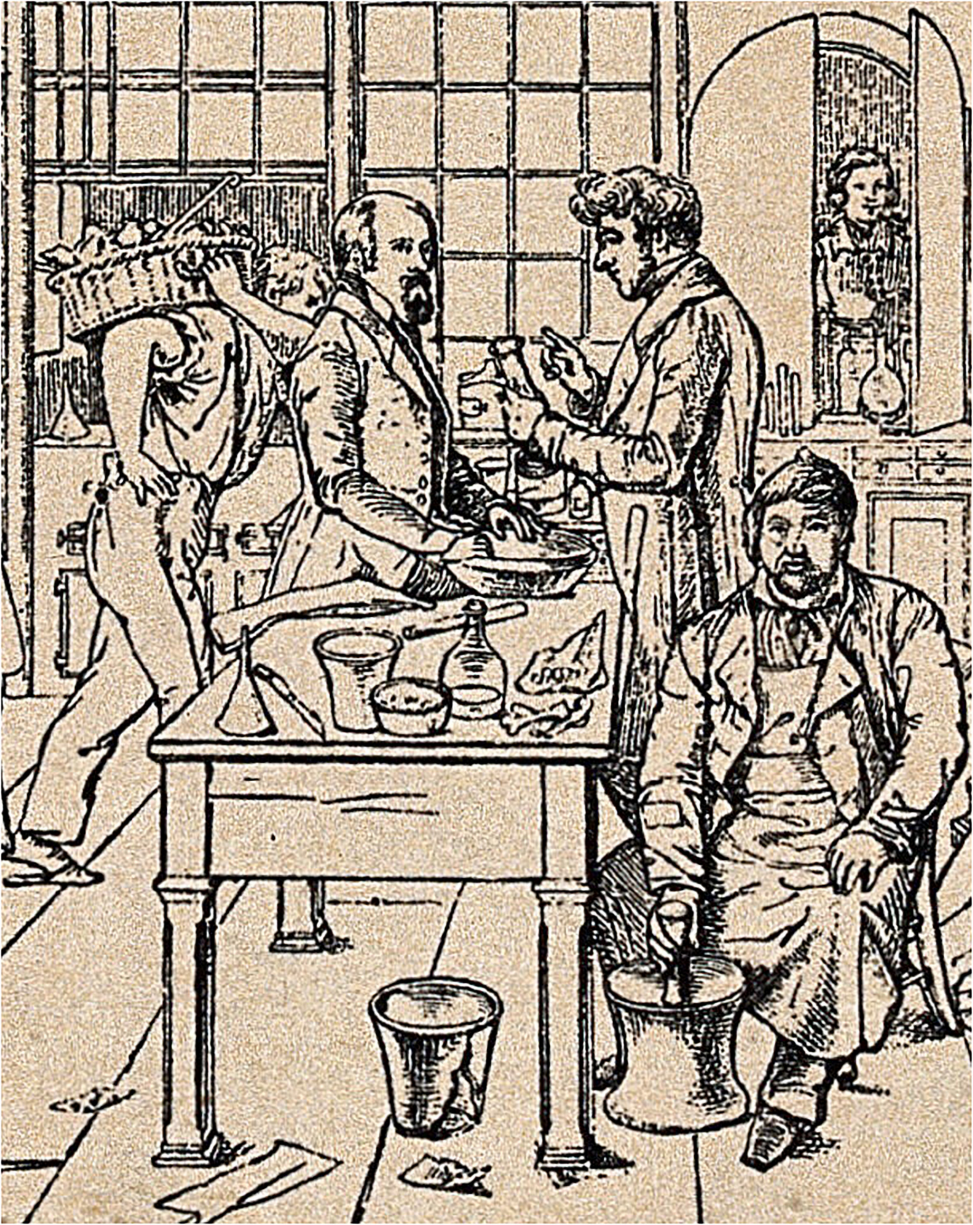
Section on the left-hand side of a larger illustration by Trautschold (1815–1877) showing students working in the chemical laboratory of Justus von Liebig in Giessen, Germany. Wilhelm Keller is depicted second from the left in discussion with Heinrich Will (1812–1890), Liebig's private assistant. Illustration provided with grateful thanks to the Wellcome Collection, London, UK. Licence: Public domain mark. Credit: ‘*Justus von Liebig and colleagues at work in the analytical laboratory in Giessen. Lithograph (?) after W. Trautschold, ca. 1840 (?)*’. See https://wellcomecollection.org/works/gem2swj7.

Wilhelm Keller attended the University of Würzburg and was also associated with the Friedrich-Wilhelms University in Berlin, graduating in chemistry during 1840 (25^th^ August) after presenting his treatise entitled, ‘*Das Bilsenkraut*’ (‘The Henbane’). The alkaloid mixtures present in extracts of henbane had long been employed in medical (and pseudo-medical) practice and the isolation of individual components was attracting increased scientific attention around this time.^13–15^ After completing his studies, he travelled to Giessen to experience further laboratory work as a student with Justus von Liebig (1803–1873). Here he was involved with other colleagues performing routine ash residue analyses of animal and plant materials, providing data for Liebig's comprehensive work on organic composition.^16^ He is captured in the 1841 illustration by the German portrait painter, Carl Friedrich Wilhelm Adolph Trautschold (1815–1877), in the middle-left of the laboratory scene in conversation with Heinrich Will (1812–1890), Liebig's private assistant, who appears to be explaining a chemical process (Figure 1).^17–19^ From his written correspondence at this time, the explicit details of his laboratory life with problems encountered in acquiring appropriate apparatus and difficulties in attaining pure starting materials, all attest to his fastidious nature and dedication and commitment to his work.^20,21^

During the latter part of 1841 (25^th^ October) he moved to Göttingen and enrolled as a medical and natural sciences student studying under the auspices of Friedrich Wöhler (1800–1882), who had himself moved from Kassel to Göttingen in the spring of 1836 to take up the position of professor of chemistry as the successor to the late Friedrich Stromeyer (1776–1835). Wöhler is often regarded as a pioneer during the emergence of organic chemistry and achieved many honours and distinctions during his lifetime.^22,23^ Keller remained at Göttingen for three years, studying a variety of subjects including anatomy and physiology as well as organic chemistry and successfully passed his doctoral examination being recognised as a physician.^20^ He appears to be the first within his family to achieve this qualification, having displayed a penchant for medical matters. A letter dated 1844 gave his address as ‘H. Keller Bookseller opposite the Russian court in Frankfurt am Main’, presumably the dwelling of his brother, August Heinrich. In mid-1844 he journeyed to Vienna where he studied pathological anatomy, ophthalmology and dermatology. Here he acquired many friendships including those of Carl von Rokitansky (1804–1878) and Joseph Škoda (1805–1881), both founding members of the modern medical school of Vienna. He then travelled via Styria (Austria) and northern Italy to Paris where he spent almost two years immersing himself in various aspects of medicine and the natural sciences.^20,21^

In 1847, for reasons unknown, perhaps the growing uprisings and revolutionary unrest in Europe, Keller decided to emigrate to America and settled in Philadelphia, living at 52 Old York road, now 5^th^ street from Green street to the Cohocksink Creek (culveted in the 1850s) at Thompson street, as confirmed by Philadelphia street directories.^24^ This was a time of massive immigration into Philadelphia,^25,26^ with its population increasing rapidly and making it the most populous city after New York.^27^ For the next eighteen months he practiced medicine but then the street directories indicate a gap in his residency in Philadelphia for 1849. This was because Keller left for Harvard College in Cambridge, Massachusetts, probably on the invitation of Louis Agassiz (1807–1873) who had been lecturing at this establishment since the mid-1840s and was later to become professor of zoology and geology. Here Keller took up an assistantship at a newly founded chemical laboratory and also gave a series of lectures on physiological chemistry and microscopy. However, this was a short-lived venture and before long he embarked on a scientific expedition to examine the flora and fauna of Lake Superior with his colleague, Agassiz, with whom he shared accommodation at Cambridge. He is mentioned with Agassiz as an ‘Instructor’ of the exploratory party in the subsequent monograph detailing their findings^28^ and as a subscriber to the publication of Agassiz's collective monograph on the natural history of the United States.^29^

In late 1850 he returned to Philadelphia and medical practice taking up successive residences at 94/134 Mulberry street, 134/112/518 Arch Street (Mulberry street was renamed Arch street in 1854) and then at 257 North Sixth street until 1862 after which (for at least five years) there is no further mention of him in street directories.^24^ In 1850, together with Heinrich Tiedemann (1813–1894), a fellow German physician born in Würzburg who had also emigrated to Philadelphia, he founded and was co-editor of ‘*Nordamerikanischer Monatsbericht für Natur- und Heilkunde*’ (North American Monthly Review of Nature and Medicine), the first German language scientific journal in America. The journal contained eight sections and covered a multitude of medical specialities. At its inauguration it acknowledged the sole assistance of a New York colleague, but later editions replaced this entry with ‘*unter Mitwirkung mehrerer Aerzte*’ (with the participation of several doctors), perhaps suggesting that input and initial enthusiasm was failing. A lack of patronage forced the journal to cease publication within a couple of years, with it only reaching the fourth volume.

After this Keller considered returning to Europe and he asked Carl Clemm-Lennig (1818–1887), a colleague from Liebig's laboratory at Giessen who had also travelled to Bridesberg, Philadelphia before returning in 1849 to Frankfurt and Mannheim, whether or not it would be favourable for him to set up as a general practitioner in Baden-Baden. This is documented in a letter dated 2^nd^ May 1853 to Karl Weltzein (1813–1870), a close colleague encountered during his time in Liebig's laboratory and who became a professor at the polytechnic school in Karlsruhe, Baden.^20,30^ Clemm-Lennig also followed this up from Mannheim with a letter directly to Weltzein, but the outcome of this particular correspondence is unclear.^20^

Keller married Augusta Maria Cramer (1834–1904) in the late 1850s. If Keller took a sojourn back to Germany for the marriage or his fiancé emigrated to America, is uncertain but there was no documented gap in his residency in Philadelphia during this period.^24^ In either case, the couple were in Philadelphia in 1859/1860. Information gleaned from the 1880 US census record of his then recent widow indicated that two of his children, Herman August Keller (1860–1912) and Friederich Harry Keller (1861–1924) were born in Philadelphia. A return to Germany then took place as several of his other children, Wilhelm Heinrich Keller (1863–1895), Franziska Ottile (Ophelia) Keller (1865–1947) and Ida Augusta Keller (1866–1932) are listed as having been born in Germany.^31,32^ The lack of continuous residency in Philadelphia after 1862 also adds weight to this assumption.^24^ Their return to Germany was presumably prompted by the growing unrest and impending outbreak of the American civil war (1861–1865). A final relocation back to Philadelphia occurred in the early 1870s when the family seemed to settle and Keller continued to practise as a physician, returning to live at 257 North Sixth street below Vine.^33^

The Proceedings of the Academy of Natural Sciences of Philadelphia (which he joined in November 1848) listed him as a member in their 1856 and 1867 decennial publications but the entry for 1877 cites his name in italics which was their way of signifying that a member had deceased.^34^ Wilhelm Keller died on 2^nd^ September 1877 at the age of 59. The cause of his demise was ‘fatty degeneration of the heart’ as cited on his death certificate. He was buried on 6^th^ September 1877 at Laurel Hill cemetery (Laurel Hill East, East Falls) on the banks of the river Schuylkill. His wife, Augusta, did not remarry and remained in America, dying on 8^th^ September 1904 in Hoboken, New Jersey. but was buried in Laurel Hill cemetery in a plot (W, 73 S½) close to her deceased husband.

## The pioneering exploration

It was during his time at Wöhler's laboratory in Göttingen that Keller became involved in the hippuric acid story. As part of his teaching method, as well as attending lectures, all of his pupils had to fulfil a period of laboratory work with Wöhler assigning specific research projects to his more advanced scholars.^35^ In response to recently published observations, Wöhler had instructed Keller, one of his students, to undertake a study on the purported conversion of benzoic acid to hippuric acid as it travelled through the human body. The results of this work were submitted for publication in *Annalen der* *Chemie und Pharmacie*, a journal established in 1832 as *Annalen der Pharmacie *with Liebig being involved in its origin and from 1838 with Wöhler as a senior editor.^36^ Liebig also published this work of his former pupil in the appendix of his *Thier Chemistry* (Animal Chemistry) which was being written at the time, thereby authoritatively disseminating these observations of Keller and Wöhler.^37^

In his paper Keller describes that he had swallowed about 32 grains (2 g) of pure benzoic acid in syrup in the evening, and then took the same dose three times during the next day. He noticed no side-effects other than profuse perspiration after the evening dose. Addition of hydrochloric acid to the subsequently collected urine, either before or after concentration, led to the deposition of a great quantity of brownish crystals. The crystals were extracted and pressed before redissolving in hot water and treated with animal charcoal. Colourless prisms an inch (2.5 cm) in length were obtained on recrystallisation. On heating they melted easily and then carbonised as the temperature increased. Under identical conditions, benzoic acid would sublime, turning directly into a vapour without passing through the liquid state. Elemental analysis (carbon content) indicated that these crystals were pure hippuric acid and allowed them to be unequivocally identified. Examining other urinary components indicated that neither urea nor uric acid were involved in its formation as had been previously intimated.^3^ The biosynthesis is now known to be an enzyme catalysed reaction between benzoic acid and the amino acid, glycine.^38^

He also suggested that as benzoic acid appeared to have no injurious effects on health, it would be possible to obtain a copious supply of hippuric acid by continuously ingesting benzoic acid thereby providing a biological means for ‘manufacturing’ the substance, a general strategy that has been exploited many times since. It is noteworthy that the initial paragraph of his paper effused that Wöhler had already expressed his opinion that during digestion benzoic acid was probably converted to hippuric acid. However, under the circumstances, this laudation of his then present mentor was probably inevitable.^39,40^ After this work, Keller remained under Wöhler's instruction and applied himself to the task of investigating the presence of cystine in urine, a sulphur-containing compound discovered several decades earlier (1810 – cystic oxide) by William Hyde Wollaston (1766–1828).

Wohler wrote about Keller's experiment in one of his many correspondences (23^rd^ June 1842) to Jöns Jacob Berzelius (1779–1848);

*Meine Vermuthung, die ich schon in einer Note bei dem Artikel Harn in der ersten Ausgabe Deiner Thierchemie ‘) aussprach, dass die Benzoesäure in dem lebenden Organismus in Hippursäure verwandelt werde, hat sich vollkommen bestätigt. Einer meiner Practicanten nahm auf meine Veranlassung mehrere Tage hintereinander jedes mal 2 Gramm Benzoesäure ein. Aus dem Harn waren dann zolllange Krystalle von reiner Hippursäure zu erhalten*. (My assumption, which I already expressed in a note in the article urine in the first edition of your ‘Animal Chemistry’, that benzoic acid is converted into hippuric acid in the living organism has been completely confirmed. At my request, one of my students took 2 grams of benzoic acid for several days in a row. Inch-long crystals of pure hippuric acid could then be obtained [*in the laboratory*] from the urine.)^41^

Berzelius responded with interest to this benzoic acid work and wrote a letter from Stockholm to Wöhler (2^nd^ August 1842) informing him of previous experimenters who had undertaken identical work;

*Ich wünsche sehr, von den Versuchen über den Einfluss der Benzoesäure auf die Bildung der Hippursäure in Kenntnis gesetzt zu werden. Du weißt wohl, dass Bouis und Ure schon vor Dir ähnUche Versuche mit demselben Resultat angestellt haben*. (I would very much like to be informed about the experiments on the influence of benzoic acid on the formation of hippuric acid. You know well that Bouis and Ure have already carried out similar experiments before you with the same result.)^41^

Wöhler had previously (at Heidelberg 1824) fed benzoic acid to a dog and (mis)identified benzoic acid in the subsequent urine, apparently showing that the compound had traversed the animal unchanged.^42^ However, on learning of Liebig's discovery of urinary hippuric acid (1829) he had contacted both Liebig and Berzelius speculating that benzoic acid was probably converted to hippuric acid during digestion.^39,40,43^ Wöhler had been the translator of the original Swedish manuscript provided by Berzelius for his *Textbook of Animal Chemistry* and had added a footnote to his own work in the section on kidneys and urine which (translated from German) read;

*It would be possible that the benzoic acid was converted into urinary benzoic acid. At least the beautiful, solid crystals of the acid which I was able to deposit in this way from the urine of a dog that had eaten benzoic acid are, in their external appearance, more consistent with urinary benzoic acid than with benzoic acid. This would also explain the presence of urinary benzoic acid in the urine of herbivorous animals, as one could assume that the benzoic acid contained in the plants in their food is converted into urinary benzoic acid during digestion*.

Particularly noticeable is the repeated use of the term ‘urinary-benzoic acid’ (*Harnbenzoësäure*), a name influenced by Berzelius, in an attempt to distinguish it from the ‘normal benzoic acid’ ingested, but there is still doubt over the certainty of identity, the term ‘hippuric acid’ (*hippursäure*} was not used.^44,45^ Wöhler became resentful and believed that his suppositions had been usurped, to be later proven and published by others. He was still bitter and aggrieved many years later when he acknowledged his misinterpretation in a letter to Berzelius (26^th^ March 1846).^41,46^

## Afterword

It appears that Keller's observations on this subject were certainly not the first to appear in the literature and he was not the first to become involved in this particular line of research, entering as it was through happenstance. However, Keller's elegant and thorough studies managed to bring clarity to this embroiled field, establishing without doubt that ingested benzoic acid was converted to hippuric acid during its journey through the human body and from this point onwards there was little if any argument. Indeed, the “vital force” (vitalism) theory, that compounds derived from biological sources possessed a special and unattainable essence, had only recently been shaken. The barriers between organic chemistry emerging in the laboratory and that occurring in living systems were only just beginning to be slowly disassembled at this time with a more comprehensive and unified system appearing.^47^

Perhaps viewing this episode as simply a step in his journey of learning he seemed to move on rapidly to other things. Liebig had offered letters of recommendation to study chemistry elsewhere and would also have liked Keller to rejoin him, but Keller seemed to harbour a displeasure at the confrontational manner in which the chemical world conducted itself and especially disliked the relationship between local professors (letter dated 9^th^ April 1844).^20^ Ultimately, his leaning towards medicine prevailed and he was to make this his career, embarking on a new life in America.
